# Taurolidine-induced Severe Anaphylaxis

**DOI:** 10.1016/j.xkme.2025.101086

**Published:** 2025-08-16

**Authors:** Lisa Christina Horvath, Gottfried Heinz, Christine Bangert, Anselm Jorda, Moritz Staudacher, Georg Gelbenegger, Bernd Jilma

**Affiliations:** 1 Department of Clinical Pharmacology, Medical University of Vienna, Vienna, Austria; 2 Department of Medicine II, Division of Cardiology, Medical University of Vienna, Vienna, Austria; 3 Department of Dermatology, Medical University of Vienna, Vienna, Austria; 4 Department of Medicine II, Division of Angiology, Medical University of Vienna, Vienna, Austria

**Keywords:** Allergic reaction, allergy, catheter lock solution, epinephrine, shock

## Abstract

Taurolidine-based catheter lock solutions are widely used for central venous catheters. Although minor infusion-related symptoms and allergic reactions have been documented, no systemic effects or severe cases of anaphylaxis have been observed. Here, we detail a case of taurolidine-induced life-threatening anaphylaxis. We report the case of a 59-year-old man who had a severe anaphylactic reaction to taurolidine following accidental intravenous infusion, which required treatment in the intensive care unit. Anaphylactic shock, which was resistant to conventional treatment with epinephrine, required additional management with norepinephrine and vasopressin. After the resolution of the anaphylactic episode, the patient was discharged from the intensive care unit. An outpatient allergy work-up using a skin prick test was performed, which confirmed a suspected allergy to taurolidine. In conclusion, taurolidine induced a severe, prolonged anaphylactic shock that did not respond to conventional treatment with intravenous and inhaled epinephrine, antihistamines, glucocorticoids, or continuous norepinephrine infusion. New treatment strategies for refractory anaphylactic shock are needed.

TauroLock is a catheter lock solution commonly used for central venous catheters because of its antimicrobial and anticoagulant effects.^1^ Although infusion-related symptoms have previously been described (erythema, facial flushing, headache, epistaxis, and nausea), no systemic effects or clinically significant abnormalities in vital signs have been reported.^2^ The onset of an allergic reaction to taurolidine in one patient was reported in a 2014 study.^3^ A 2022 retrospective analysis reported 6 allergic reactions to taurolidine in 470 patients during 700,232 catheter days, all of them were medically significant but not immediately life-threatening.^4^ This case report describes a previously unreported life-threatening anaphylactic reaction to taurolidine.

## Case Report

A 59-year-old man with a history of short bowel syndrome was admitted to the ward because of a central venous catheter (Hickman catheter) associated infection caused by *Enterococcus faecium*. The patient's medical history included a severe form of familial adenomatous polyposis requiring multiple surgical procedures resulting in short bowel syndrome. The catheter infection was initially treated with piperacillin/tazobactam and daptomycin. This was switched to ampicillin after pathogen identification in blood cultures. The patient’s Hickman catheter was removed and subsequently replaced after the infection had subsided. The new catheter was sealed using a taurolidine containing catheter lock solution (TauroLock, Taurolock GmbH).

On day 15 of hospitalization, the catheter lock solution was not aspirated but mistakenly flushed before catheter usage. Shortly afterward, the patient experienced dyspnea and generalized erythema. As an allergic reaction was suspected, fluids, 60-mg diphenhydramine, 250-mg prednisolone, and a single dose of 100-μg epinephrine were given intravenously. On arrival of the medical emergency team, the patient was awake (Glasgow Coma Scale 10), appeared to be in respiratory distress, with a peripheral oxygen saturation of 70%, no apparent stridor, tachycardia (150 bpm) and a nonmeasurable noninvasive blood pressure. After oxygen delivery via mask, abundant volume resuscitation and inhaled epinephrine (1,000 μg), his peripheral oxygen saturation remained inadequate (90%) and he required advanced treatment in the intensive care unit (ICU) (Table 1).

**Table 1:** Patient Characteristics and Laboratory Findings at Initial Presentation and Over Time

Medical History	Familial adenomatous polyposis, multiple surgical procedures resulting in short bowel syndrome, central venous catheter (Hickman catheter) associated infection, chronic kidney disease stage 3B, hepatic steatosis					
General Condition	Awake, GCS 10 and responsive, but in respiratory distress					
Concomitant Medication	Pregabalin; pancreatin; vitamins B1, B6, and B12; mirtazapine, loperamide, potassium-chloride, magnesium, pantoprazole, racecadotril, teduglutide, levocetirizine, hydromorphone, ampicillin, glucose-1-phosphate, metamizole, ondansetron					
Vital Signs	Noninvasive blood pressure nonmeasurable, heart rate 150 bpm, peripheral O_2_ saturation 70%					
Laboratory findings BGA arterial (normal range)	Initial	1 h	5 h	6 h	9 h	12 h
pH (7.35-7.45)	7.19	7.6	7.35	7.36	7.4	7.42
pCO_2_ (35-48 mm Hg)	31.0	34.9	32.2	29.9	34.8	36.1
pO_2_ (83-108 mm Hg)	166	98.9	120	-	102.0	89.4
Base excess (0 ± 2 mmol/L)	–15.1	–10.6	–7.3	–8.0	–3.2	–1.1
Bicarbonate (24-31 mmol/L)	13.0	16.1	18.8	18.4	22.1	23.7
Lactate (<1.8 mmol/)L	7.2	6.7	6.4	5.7	3.1	2.0

Abbreviations: BGA, blood gas analysis; GCS, Glasgow Coma Scale.

**Figure 1:**
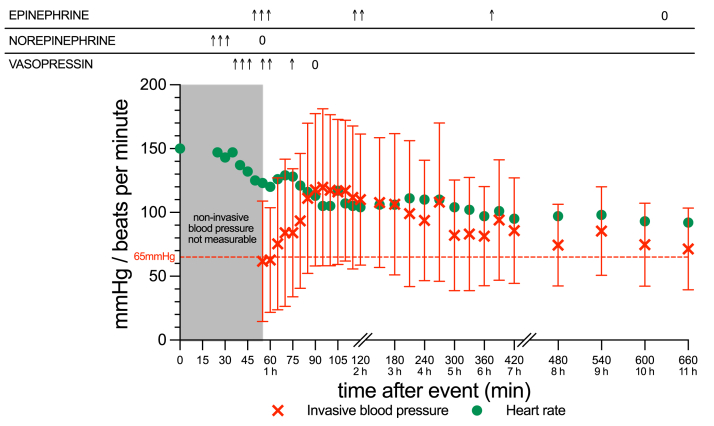
Blood pressure and heart rate over time. The figure shows the patient’s blood pressure and heart rate over time (green dots denote the heart rate, red lines denote the systolic and diastolic blood pressure and mean arterial pressure). From onset of anaphylaxis and arrival of the medical emergency team up to 55 minutes (time of successful placement of an arterial line), no noninvasive blood pressure could be measured (gray-marked area). During this time period, the patient was blurred but responsive to pain. The placement of an arterial line was delayed because of a difficult vascular anatomy. After placement, continuous norepinephrine was stopped and replaced by epinephrine and vasopressin, which led to intermittent blood pressure overcorrection. Continuous epinephrine and vasopressin could be successfully weaned within the next hours. Arrows (↑) denote a semiquantitative measurement of catecholamine doses. Given the emergency circumstance, the initial catecholamine doses were not accurately documented.

On arrival in the ICU, the patient’s noninvasive blood pressure was still unmeasurable, and placement of an arterial line and central venous catheter were prolonged. Continuous norepinephrine was started peripherally until placement of a central venous line and was then switched to continuous epinephrine and vasopressin (Fig 1). The first recorded invasive blood pressure measured at 55 minutes after the taurolidine application was 113/18 mm hg (mean arterial pressure 50) as compared with a resting blood pressure of 130/80 at the ward. Initial venous blood gas analysis showed combined metabolic and respiratory acidosis (pH 7.09; pCO_2_, 58 mm hg; base excess, –11.3; lactate level, 8.5 mmol/L). Peripheral oxygen saturation remained stable under oxygen insufflation without the need of further respiratory support. Under continued volume resuscitation (total 6,000 mL), intravenous vasopressors could be successfully weaned within the following 10 hours and the patient was transferred back to the ward the next day. Four months later, the patient underwent an allergy work-up with extensive skin testing (prick and intracutaneous tests using dilution series) that confirmed an allergy to taurolidine (Table S1 and Fig S1).

## Discussion

The patient was allergic to buprenorphine and had a presumptive contrast media allergy that could not be confirmed on recent radiologic investigations. He received a tunneled catheter for hemodialysis in 2016, which was flushed and blocked with a taurolidine solution. Careful medical history showed that a first reaction to a taurolidine solution had occurred with dyspnea and pruritus about 2 years before the current event, and a second reaction with dyspnea, pruritus, flush, and edematous eyelids occurred 1 year prior that required urgent care by prehospital emergency medical services including an emergency physician. Since then, the patient himself had decided to no longer use TauroLock, but unfortunately a proper diagnosis or documentation of an allergy to Taurolock was not attempted. Although discontinuation of the culprit drug was intuitive, it may also have reduced tolerance to the drug before the third life-threatening event of protracted anaphylaxis.

This case illustrates the occurrence of prolonged anaphylactic shock resistant to repeated and continuous administration of catecholamines, including epinephrine and norepinephrine. Hemodynamics stabilized only after continuous infusion of epinephrine and vasopressin, which have been suggested as therapeutic option in the case of epinephrine-resistant anaphylaxis.^5^

The high vasopressor demand and volume resuscitation confirm that conventional catecholamines work poorly at least in some patients with anaphylactic shock.^6^ Intramuscular epinephrine is recommended as first-line treatment for anaphylactic shock; unfortunately, there is no proof for efficacy from controlled trials in human anaphylaxis. In contrast, animal trials in dogs and own ongoing studies in guinea pigs raise serious doubts that bolus administration of epinephrine may be effective.^7^

Histamine concentrations increase >100-fold in grade 4 anaphylaxis induced by hymenoptera stings, or radiocontrast media, whereas antihistamines only protect against minor 2- to 3-fold elevations of plasma histamine.^8^, ^9^, ^10^ Glucocorticoids do not work fast enough to be of any use in the treatment of acute anaphylaxis. The urgent need for more specific, more effective and fast acting treatments of anaphylaxis may be met by future clinical development of a recombinant human diamine oxidase.^11^

In summary, taurolidine caused protracted anaphylactic shock, refractory to intravenous and inhaled epinephrine, adjunctive therapy with antihistamines and glucocorticoids, and continuous infusion of norepinephrine.
