# Supplementary material for: Taurolidine-induced Severe Anaphylaxis
Source: Kidney Med. 2025 Aug 16;7(10):101086. doi: 10.1016/j.xkme.2025.101086 (PMC12495460; doi:10.1016/j.xkme.2025.101086)
Supplement: Supplementary File (PDF) — Figure S1; Table S1. [file mmc1.pdf]

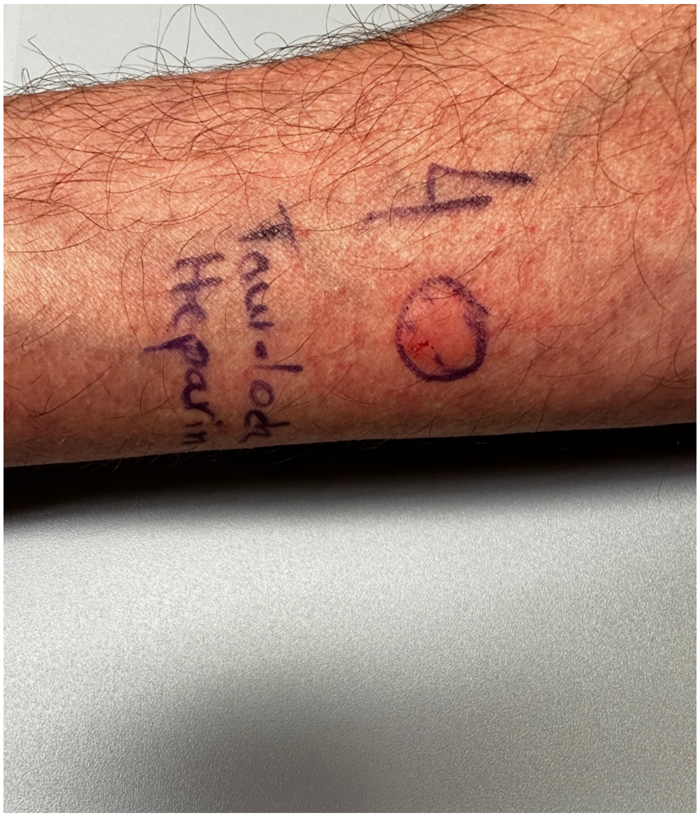

Figure S1. Intracutaneous test showing a skin reaction to TauroLock^{TM} -Heparin.

Table S1. Skin testing (prick and intracutaneous tests using dilution series)

Test substance	Prick test	Intracutaneous 1:250	Intracutaneous 1:25	Intracutaneous 1:5
**Heparin**	negative	negative (200 I.E/mL)	X	X
**Taurolock–Heparin**	negative	negative	negative	**positive**
**Taurolock–Urokinase**	negative	negative	**positive**	**positive**
